# Plastic and evolutionary responses to climate change in fish

**DOI:** 10.1111/eva.12135

**Published:** 2014-01-02

**Authors:** Lisa G Crozier, Jeffrey A Hutchings

**Affiliations:** 1 Northwest Fisheries Science CenterSeattle, WA, USA; 2 Department of Biology, Dalhousie UniversityHalifax, NS, Canada; 3 Department of Biosciences, Centre for Ecological and Evolutionary Synthesis, University of OsloOslo, Norway

**Keywords:** adaptation, climate change, evolutionary theory, fisheries management, life-history evolution, phenotypic plasticity

## Abstract

The physical and ecological ‘fingerprints’ of anthropogenic climate change over the past century are now well documented in many environments and taxa. We reviewed the evidence for phenotypic responses to recent climate change in fish. Changes in the timing of migration and reproduction, age at maturity, age at juvenile migration, growth, survival and fecundity were associated primarily with changes in temperature. Although these traits can evolve rapidly, only two studies attributed phenotypic changes formally to evolutionary mechanisms. The correlation-based methods most frequently employed point largely to ‘fine-grained’ population responses to environmental variability (i.e. rapid phenotypic changes relative to generation time), consistent with plastic mechanisms. Ultimately, many species will likely adapt to long-term warming trends overlaid on natural climate oscillations. Considering the strong plasticity in all traits studied, we recommend development and expanded use of methods capable of detecting evolutionary change, such as the long term study of selection coefficients and temporal shifts in reaction norms, and increased attention to forecasting adaptive change in response to the synergistic interactions of the multiple selection pressures likely to be associated with climate change.

## Introduction

Anthropogenic climate change^1^ is one of the most important threats to global biodiversity over the next century (Sala et al. 2000; Thomas et al. 2004; Lovejoy and Hannah 2005). Now that ‘Warming of the climate is unequivocal’ (IPCC 2013), how are biological systems reacting? A rich literature documents *potential* evolutionary and plastic responses to physical drivers in fish. However, predicting *actual* responses in natural populations remains a core challenge because observed responses typically fail to match predictions based on theory or laboratory experiments (Merilä et al. 2001). To clarify what patterns have been observed, we undertook a specific review of the literature that detected phenotypic responses to climate change in wild fish populations, with a particular interest in the extent to which these responses could be attributed to evolutionary or plastic processes.

To anticipate the phenotypic consequences of climate change in fish, we can draw on a vast literature (for reviews, see Brett 1956; Fry 1967; Brett 1995; Wootton 1998; Walters and Martell 2004; Rijnsdorp et al. 2009). This volume of work stems from the high economic and cultural value of fisheries globally, fish farming and hatcheries, the aquarium trade, and use of fish as model systems in developmental genetics and disease research. The concept of fisheries-induced evolution, initiated primarily in the late 1970s/early 1980s (Handford et al. 1977; Ricker 1981), generated numerous analyses to evaluate the magnitude and likelihood of this form of evolutionary change (reviewed by Dieckmann and Heino 2007; Hutchings and Fraser 2008). Over roughly the same period of time, the salmon aquaculture industry and the salmonid hatchery ‘industry’ generated numerous papers pertaining to selection responses, trait heritability, temperature effects on myriad characteristics and genetic differentiation (e.g. references cited by Mousseau and Roff 1987; Purdom 1993). Armed with significantly enhanced (relative to most other taxa) empirically and financially rewarding research opportunities, fish geneticists, population biologists, ecologists and evolutionary biologists have made considerable advances in our knowledge of adaptation, selection responses, and rates of evolutionary change in fishes (e.g. Carlson et al. 2004; Hendry and Stearns 2004; Barrett et al. 2011). However, the profoundly intertwined mechanisms of evolution and plasticity in most climate-sensitive traits presents a major challenge for detecting adaptation to climate change in natural populations.

## Reaction norms and acclimation

Phenotypic responses (e.g. growth rate, timing of reproduction) to environmental conditions, especially temperature, are routinely plastic in nature, but genetic variability differentiates the plastic response among families within populations, among populations and between species (e.g. Haugen and Vøllestad 2000; Jensen et al. 2008; Baumann and Conover 2011; Hutchings 2011). Reaction norms, graphical representations of phenotypic change along an environmental gradient (Scheiner 1993; Schlichting and Pigliucci 1998; Hutchings et al. 2007), constitute a standard means of describing plasticity. Evidence of genetic differentiation, and possible adaptation, appear through differences in the shape, intercept and (or) slope of reaction norms (Lande 2009; Chevin et al. 2010).

Plasticity in stress tolerance usually takes the form of ‘acclimation’, in which a history of exposure to particular conditions changes an organism's response to a challenge (Angilletta 2009; Kassahn et al. 2009). Because climate change involves prolonged exposure to altered conditions, acclimation will presumably play a key role in effecting phenotypic changes (Stillman 2003; Hofmann and Todgham 2010). Experiments measuring stress tolerance typically expose all individuals to a common rearing environment, attempting to control for acclimation responses (Beitinger et al. 2000; Johansen and Jones 2011). Variation in the extent to which acclimation alters performance can, in some instances, reflect local adaptation, as evidenced by Antarctic fishes that experience unusually constant temperatures (Bilyk and DeVries 2011). However, the conditions necessary to trigger an acclimatory response differ among species, complicating full characterization of this response by experimental methods. For example, prolonged warm acclimation enhances high-temperature tolerance in killifish (*Fundulus heteroclitus*), but repeated heat shocks do not (Healy and Schulte 2012). In zebrafish (*Danio rerio*), developmental plasticity affects acclimation to temperature substantially later in life (Scott and Johnston 2012). Similarly, developmental conditions can affect reaction norms for growth in Atlantic cod, *Gadus morhua* (Hurst et al. 2012). Thus, short-term exposure to acclimatory conditions during later life stages might underestimate the full acclimation potential of some species. At the extreme, a full generation might be necessary to trigger acclimation responses, as shown in the tropical damselfish *Acanthochromis polyacanthus* (Donelson et al. 2012) and sheepshead minnows *Cyprinodon variegatus* (Salinas and Munch 2012).

## Are these responses adaptive?

There is some evidence that genetic changes in phenotypically plastic responses to temperature can be adaptive. One notable example in freshwater fish pertains to Norwegian populations of grayling (*Thymallus thymallus*). Although they once shared a common ancestor, the populations have been reproductively isolated from one another and exposed to different environments for more than 15 generations. Based on the results of a common-garden experimental protocol, this timeframe was sufficient to allow for population differences to emerge in plastic responses of several early-life traits to temperature (Haugen and Vøllestad 2000). Populations in colder lakes developed a more cold-adapted reaction norm for growth, including better growth at cooler temperatures and more efficient conversion from yolk to body mass compared with populations from warmer lakes (Kavanagh et al. 2010). These responses show a signature of selection as opposed to genetic drift (Qst > Fst), and these patterns correlated with lake temperature rather than physical distance.

Arguments in favour of the hypothesis that population differences in thermal reaction norms represented adaptive responses to local environments were based on observations that the traits examined were closely linked to fitness and that survival was highest at the temperatures that they were most likely to experience in the wild (a similar approach was adopted by Hutchings et al. 2007 in their reaction-norm study in Atlantic cod). The hypothesis that genetic variation in plasticity represents adaptive responses to different thermal regimes in early life is also supported by the discovery of temperature-associated SNPs (single nucleotide polymorphisms) in Atlantic cod that appear to be under selection (Bradbury et al. 2010, 2013). A particularly interesting example of genomic thermal plasticity in fish was reported by Croisetière et al. (2010) in brook trout (*Salvelinus fontinalis*). They found that the way in which the expression of the MHC classII*β* gene changes with temperature is associated with the basepair length of an associated temperature-sensitive mini-satellite, which may suggest a genomic underpinning for thermal plasticity. Immune-relevant genes in general follow latitudinal clines that are correlated with temperature (Dionne et al. 2007; Tonteri et al. 2010).

Common-garden experiments have featured prominently in many studies of the adaptive significance of population differences in phenotypic responses to temperature (Franks et al. 2013). Particularly relevant examples in fish include those of countergradient variation, which occurs when genetic differences counteract environmental effects, reducing phenotypic variation between populations; it is expected when stabilizing selection favours similar phenotypes in different environments (Conover and Schultz 1995). In fishes, evidence that countergradient variation reflects adaptation to thermal environments exists for some species – for example, Atlantic silversides, *Menidia menidia* (Conover and Present 1990) and Atlantic cod (Marcil et al. 2006) but not necessarily others – for example, mummichog, *Fundulus heteroclitus* (Fangue et al. 2009).

Given the genetic variability in temperature responses documented in many fish species (Beitinger et al. 2000), coupled with persuasive evidence in support of the hypothesis that phenotypic responses to temperature can be adaptive, it seems highly probable that many fish possess sufficient additive genetic variability to respond adaptively to climate change, although our review reveals only limited evidence of this to date.

## Primary physical impacts of climate change relevant for fishes

The two primary physical drivers of climate change in the ocean are rising ocean temperature and carbon dioxide absorption (Hoegh-Guldberg and Bruno 2010; Gruber 2011; Hale et al. 2011; Koehn et al. 2011; Doney et al. 2012; Gruber et al. 2012; Collins 2014; Reusch 2014). These drivers have clearly changed over the past century in response to rising greenhouse gas emissions (IPCC 2007; Blunden and Arndt 2013; NCADAC 2013). Effects of warming oceans cascade beyond temperature change alone to alter Arctic ice volume, sea level and hence coastal habitat quality and quantity, salinity, vertical stratification, weather (i.e. precipitation, storm intensity and wind; Francis and Vavrus 2012; Liu et al. 2012), ocean current circulation and hypoxia (i.e. low oxygen levels; IPCC 2007). Carbon dioxide absorption interacts with many of these thermally induced phenomena to increase exposure to corrosive and hypoxic water. Rates of change vary geographically and in some cases local processes reverse the global trends (e.g. more intense wind sheer can increase coastal upwelling of deep water, which reduces local temperatures because deep water is much cooler than surface water). Forecasted large-scale changes in ocean circulation patterns are uncertain, as are consequences for multidecadal oscillations in climate, as reflected by indices such as the North Atlantic Oscillation (NAO), the Pacific Decadal Oscillation (PDO) and El Niño-Southern Oscillation (ENSO).

In fresh water, the primary drivers of climate change include rising water temperature, altered hydrological regimes (i.e. the timing of flows of different magnitudes), thermal stratification, decreased dissolved oxygen and increased toxicity of pollutants (Ficke et al. 2007; Stoks et al. 2014; Urban 2014). Hydrological regimes are in transition in regionally specific ways, including shifts in the magnitude and timing of floods, increasingly intense droughts and heat waves (IPCC 2012). More frequent and more intense precipitation events have numerous consequences, including added run-off of pollutants and nutrients into the water, increasing sediment load and eutrophication. These inputs can reduce the quality of fish habitat and result in harmful algal blooms and hypoxic ‘dead zones’ (NCADAC 2013). Loss of water, due to increased water vapour in the air and competition with humans, will affect groundwater, aquifers and wetlands. Many cold-water fish are expected to move or contract their ranges to higher elevation (Wenger and Olden 2012), while warm-water invasive species expand their ranges (Rahel and Olden 2008; Al-Chokhachy et al. 2013). Key fish habitats, such as coral reefs, mangrove and kelp forests, will likely decline (Hoegh-Guldberg and Bruno 2010).

## Natural climatic fluctuations

**Figure 1:**
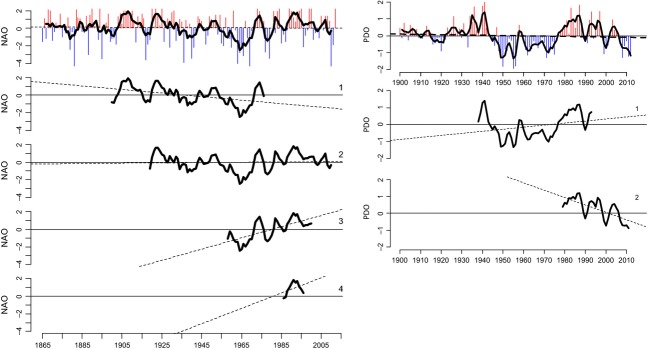
a Left: The North Atlantic Oscillation (NAO) station-based seasonal index for winter (includes December – February) since 1865 (Hurrell 1995): annual index (vertical lines) are shown in the top panel, and 5 year running mean (black line) is shown in all panels. The portion of the NAO time series in each lower panel show the years included in a selection of studies in Table 1: (i) Sundby and Nakken (2008); (ii) Rogers et al. (2011); (iii) Beaugrand et al. (2003); (iv) Kjesbu et al. (1998). Dotted lines are linear regression lines fit to the period of data shown. Right: The PDO index since 1900: annual index (vertical lines) are shown in the top panel, and 3 year running mean (black line) is shown in all panels. The portion of the PDO time series in each lower panel show the years included in selected studies from Table 1: (i) Quinn and Adams (1996); (ii) Kovach et al. (2012). Dotted lines are linear regression lines fit to the period of data shown. Note that local temperatures do not necessarily follow the PDO, but may have additional trend upon them, as in the Kovach study.

Although many recent environmental trends are consistent with anthropogenic climate change, these trends might not continue in a linear fashion. Short-term trends easily nest within longer climate cycles, and generally ecological studies encompass only parts of these cycles. Figure 1 shows the NAO and PDO indices for 100–150 years and the portions of these time series captured by the studies listed in Table 1. The trend lines fit to the shorter time series show a range of slopes from negative to positive, depending on which phase of the larger oscillation coincided with the study. Although local temperatures do not track the larger climate indices perfectly, there are clear signals of these oscillations across continents as well as the ocean (Mantua et al. 1997; Stenseth and Mysterud 2005). For example, more streams in the western United States have cooled than warmed since 1987 (Arismendi et al. 2012). Warming trends predominate when records stretch back to 1950 (Arismendi et al. 2012), consistent with PDO cycling (Fig. 1).

**Table 1:** Environmental drivers and temporal coverage of the studies of phenotypic change

Species	Location	Years	# Years	Reference	Trait	Driver
Atlantic salmon (*S. salar*)	Norway	1991–2005	14	Otero et al. (2012)	Age at maturity	SST
Atlantic salmon (*S. salar*)	Scotland	1975–2010	35	Todd et al. (2012)	Age at maturity	Stream T
Atlantic salmon (*S. salar*)	River Imsa, Norway	1976–2001	25	Juanes et al. (2004)	Age at maturity	NAO
Cod (*G. morhua*)	North Atlantic	1943–1999	56	Ottersen et al. (2006)	Age at maturity	SST
Sockeye salmon (O. nerka)	Fraser River, Canada	1952–1993	42	Cox & Hinch, (1997)	Age at maturity	SST
Atlantic salmon (*S. salar*)	Scotland	1975–2010	35	Todd et al. (2012)	Age at smolting	Stream T
Atlantic salmon (*S. salar*)	31 stocks N. Am & Eur	1989–2009	20	Russell et al. (2012)	Age at smolting	
Eurasian ruffe (*G. cernuus*)	Estonia	1951–1998	47	Ahas and Aasa (2006)	Appearance	Air T, NAO
European perch (*Perca fluviatilis*)	Estonia	1951–1998	47	Ahas and Aasa (2006)	Appearance	Air T, NAO
Cod (*G. morhua*)	Arcto-Norwegian region	1900–1976	76	Sundby and Nakken (2008)	Fecundity	SST
Cod (*G. morhua*)	Barents Sea	1986–1996	10	Kjesbu et al. (1998)	Fecundity	SST-capelin
Atlantic salmon (*S. salar*)	Scotland & Canada	1964–1993	29	Friedland et al. (2005)	Growth	SST
Atlantic salmon (*S. salar*)	NE Atlantic	1992–2006	14	Todd et al. (2008)	Growth	SST
Cod (*G. morhua*)	Gulf of Alaska	2006–2008	3	Hurst et al. (2012)	Growth	SST
Herring (*Clupea harengus*)	Thames estuary, UK	1977–1992	16	Attrill and Power (2002)	Growth	NAO
Plaice (*Pleuronectes platessa*)	North Sea	1970–2004	34	Teal et al. (2008)	Growth	SST
Smelt (*Osmerus eperlanus*)	Thames estuary, UK	1977–1992	16	Attrill and Power (2002)	Growth	NAO
Sole (*Solea solea*)	North Sea	1970–2004	34	Teal et al. (2008)	Growth	SST
Sprat (*Sprattus sprattus*)	Thames estuary, UK	1977–1992	16	Attrill and Power (2002)	Growth	NAO
Whiting (*Merlangius merlangus*)	Thames estuary, UK	1977–1992	16	Attrill and Power (2002)	Growth	NAO
Cod (*G. morhua*)	Barents Sea	1958–2000	42	Beaugrand et al. (2003)	Juvenile survival	SST–copepods
American shad (*Alosa sapidissima*)	Columbia River, US	1938–1993	55	Quinn and Adams (1996)	Migration timing (A)	Stream T
Atlantic salmon (*S. salar*)	NE US & SE Canada	1978–1999	21	Juanes et al. (2004)	Migration timing (A)	Stream T
Atlantic salmon (*S. salar*)	Dalälven River	1960–2002	42	Dahl et al. (2004)	Migration timing (A)	SST & stream T
Atlantic salmon (*S. salar*)	Asturian Rivers, Spain	1956–2006	50	Valiente et al. (2011)	Migration timing (A)	Air T & NAO
Brown Trout (*S. trutta*)	Dalälven River	1960–2002	42	Dahl et al. (2004)	Migration timing (A)	SST & stream T
Cutthroat trout (*O. clarkii clarkii*)	Auke Creek, Alaska	1970–2010	40	Kovach et al. (2013)	Migration timing (A)	Stream T
Dolly Varden char (*S. malma*)	Auke Creek, Alaska	1970–2010	40	Kovach et al. (2013)	Migration timing (A)	Stream T
Flounder (*Platichthys flesus*)	UK	1953–1965	13	Sims et al. (2004)	Migration timing (A)	SST, NAO
Pink salmon (*O. gorbuscha*)	Auke Creek, Alaska	1979–2011	32	Kovach et al. (2012)	Migration timing (A)	Stream T
Pink salmon (*O. gorbuscha*)	Auke Creek, Alaska	1972–2005	33	Taylor (2008)	Migration timing (A)	Stream T
Sockeye salmon (*O. nerka*)	Columbia River, US	1949–1993	44	Quinn and Adams (1996)	Migration timing (A)	Stream T
Sockeye salmon (*O. nerka*)	Columbia River, US	1949–2005	56	Crozier et al. (2011)	Migration timing (A)	Stream T & flow
Atlantic salmon (*S. salar*)	Northern Ireland	1978–2008	30	Kennedy and Crozier (2010)	Migration timing (J)	Stream T
Atlantic salmon (*S. salar*)	62 stocks N. Am & Eur	Variable		Russell et al. (2012)	Migration timing (J)	
Pink salmon (*O. gorbuscha*)	Auke Creek, Alaska	1972–2005	33	Taylor (2008)	Migration timing (J)	Stream T
Coho salmon (*O. kisutch*)	Auke Creek, Alaska	1970–2010	40	Kovach et al. (2013)	Migration timing (J,A)	Str T, flow & SST
Pink salmon (*O. gorbuscha*)	Auke Creek, Alaska	1970–2010	40	Kovach et al. (2013)	Migration timing (J,A)	Stream T
Sockeye salmon (*O. nerka*)	Auke Creek, Alaska	1970–2010	40	Kovach et al. (2013)	Migration timing (J,A)	Stream T & flow
Bass (*Dicentrarchus labrax*)	Thames estuary, UK	1977–1992	16	Attrill and Power (2002)	Size	NAO
Cod (*G. morhua*)	Norway	1919–2010	91	Rogers et al. (2011)	Size	SST
Dab (*Limanda limanda*)	Thames estuary, UK	1977–1992	16	Attrill and Power (2002)	Size	NAO
Flounder (*Platichthys flesus*)	Thames estuary, UK	1977–1992	16	Attrill and Power (2002)	Size	NAO
Plaice (*Pleuronectes platessa*)	Thames estuary, UK	1977–1992	16	Attrill and Power (2002)	Size	NAO
Smelt (*Osmerus eperlanus*)	Thames estuary, UK	1977–1992	16	Attrill and Power (2002)	Size	NAO
Sockeye salmon (*O. nerka*)	SW Alaska	1962–2002	40	Schindler et al. (2005)	Size	Ice out
Sole (*Solea solea*)	Thames estuary, UK	1977–1992	16	Attrill and Power (2002)	Size	NAO
Bream (*Abramis brama*)	Estonia	1951–1990	39	Noges and Jarvet (2005)	Spawn timing	Stream T
Burbot (*Lota lota*)	Estonia	1951–1998	47	Ahas and Aasa (2006)	Spawn timing	Air T, NAO
Eurasian dace (*Leuciscus cephalus*)	Estonia	1951–1998	47	Ahas and Aasa (2006)	Spawn timing	Air T, NAO
Eurasian ruffe (*G. cernua*)	Estonia	1951–1998	47	Ahas and Aasa (2006)	Spawn timing	Air T, NAO
European perch (*Perca fluviatilis*)	Estonia	1951–1998	47	Ahas and Aasa (2006)	Spawn timing	Air T, NAO
Northern pike (*Esox lucius*)	Estonia	1951–1998	47	Ahas and Aasa (2006)	Spawn timing	Air T, NAO
Roach (*Rutilus rutilus*)	Estonia	1951–1990	39	Noges and Jarvet (2005)	Spawn timing	Stream T
Roach (*Rutilus rutilus*)	Lake Geneva, France	1983–2000	18	Gillet and Quétin (2006)	Spawn timing	Lake T
Smelt (*Osmerus eperlanus*)	Estonia	1951–1998	47	Ahas and Aasa (2006)	Spawn timing	Air T, NAO
Walleye (*Sander vitreus*)	12 populations, Minnesota, US		Variable	Schneider et al. (2010)	Spawn timing	Ice out

Species genera: *S*. *Salmo*, O. *Oncorhynchus, G. cernua: Gymnocephalus, G. morhua: Gadus*. Trait: A, adult, J, juvenile. Driver: T, temperature, SST, sea surface temperature.

If the variability of the NAO or PDO increases, subperiod trends will be steeper and persist longer. Although the impact of anthropogenic climate change on the variability in these oscillations is uncertain (NCADAC 2013), some authors have argued that changes are already apparent. First, a long-term linear trend in global temperature overlays the oscillations (Fig. 1 in Klyashtorin et al. 2009). When this trend is removed, the ∼60-year cycle is quite apparent (Fig. 2 in Klyashtorin et al. 2009). The impact of the trend is most pronounced at the peaks of the oscillation but could be detected at any point if the natural cycle is accounted for. Second, the intensity of the periodicity has increased during the last millennium, reaching a peak at the end of the twentieth century (Klyashtorin et al. 2009). Climate change might be affecting the range (peak to trough) of these cycles (Goodkin et al. 2008).

**Figure 2:**
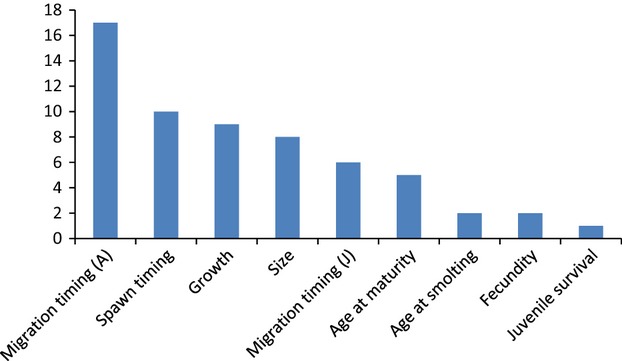
Frequency distribution of traits showing shifts that correlate with environmental drivers in recent decades. Migration timing is broken into adult migrations (A), which are usually spawning migrations (includes “appearance” in Table 1) or seawater to freshwater migrations, and juvenile (J) or freshwater to saltwater migrations.

## Proximate drivers and traits likely to respond to climate change

Ecological impacts of these physical changes will vary by species and location. Physiologically, rising temperatures have some nearly universal effects in fish, such as increasing metabolic rates (Fry 1967). However, the ecological and evolutionary consequences of rising temperature depend on many factors, including population-specific proximity to lethal limits or growth optima (Pörtner and Peck 2010; Somero 2010), interspecific dynamics (Finstad et al. 2011) and disease impacts (Marcos-Lopez et al. 2010). Behavioural responses can reduce expression of physiological responses through thermoregulation (Angilletta 2009), or increase them because of competing pressures such as disease infection (Landis et al. 2012). Multiple stressors might act additively or synergistically (e.g. with compounding effects) and thus need to be considered in any examination of phenotypic shifts. Nonetheless, most research has focused on single factors.

## Direct stress (temperature, hypoxia, pCO_2_, diseases)

The most straightforward proximate driver of global change in fish is direct physiological stress due to various factors − such as lowered pH, lowered oxygen levels, rising temperature − which leads secondarily to increased disease prevalence and morbidity in many cases (Rijnsdorp et al. 2009). Organisms integrate multiple stressors physiologically, accumulating factors that limit oxygen metabolism and aerobic scope (Pörtner et al. 2001; Pörtner and Farrell 2008; Anttila et al. 2013). Tolerance of these conditions has a strong genetic basis and shows high levels of local adaptation (Côté et al. 2012; Donelson and Munday 2012; Madeira et al. 2012; Munday et al. 2012), and thus, these traits clearly evolve by natural selection. Although most studies have focused on heat tolerance, because many fish communities face prolonged winter periods in temperate and high latitude areas, specialized adaptations for tolerating winter will also face a changing selection regime (Pörtner and Peck 2010; Shuter et al. 2012). Many other traits show temperature sensitivity and might threaten population viability, such as sex determination, sexual abnormalities and fertility (Strussmann et al. 2010; Pankhurst and Munday 2011). However, the use of latent genetic variation in local adaptations, such as development time in rainbow trout (*Oncorhynchus mykiss*), suggests evolution in key traits could occur quickly (Miller et al. 2012).

Selection for disease tolerance will likely intensify because warmer environments exhibit a general increase in the diversity of diseases, increased population growth rates of most microorganisms (Macnab and Barber 2012) and increased vulnerability of coldwater fishes. Furthermore, ongoing human activity tends to spread pathogens (Harvell et al. 1999; Marcos-Lopez et al. 2010). Historically, fish have adapted to high disease loads in warmer environments by enhancing the diversity of Major Histocompatibility Complex (MHC) genes (Dionne et al. 2007; Bowden 2008; Marcos-Lopez et al. 2010) and local adaptation to specific diseases (Beacham and Evelyn 1992; Bartholomew 1998).

Impacts of ocean acidification on fish are less well understood than those associated with temperature (Kroeker et al. 2010; Denman et al. 2011; but see examples of phytoplankton evolution in Reusch 2014). High pCO_2_ affects fish physiology directly, through developmental exposure (Franke and Clemmesen 2011; Frommel et al. 2012), olfaction (Munday et al. 2009; Dixson et al. 2010), and a variety of behaviours such as settlement and the avoidance of predators (Munday et al. 2012). Fish sensitivity also includes vulnerability to habitat loss (Gruber 2011; Gruber et al. 2012) and prey availability because of difficulties for skeleton- or shell-forming organisms under lower calcium-carbonate saturation states (Orr et al. 2005; Heath et al. 2012). Marine viruses interact with ocean biogeochemical cycles and fish dynamics in ways that are currently unpredictable but may profoundly influence ocean ecosystems (Danovaro et al. 2011).

## Food-web dynamics affect behaviour, growth and survival

Although direct physical stressors are clearly limiting on some level, the primary mechanism cited in the literature by which climate impacts fish population dynamics involves the food web. Physical oceanographic, hydrological and limnological drivers determine the geographical distribution, total abundance, species composition and physiological condition of phytoplankton, zooplankton and plants (Collins 2014; Reusch 2014), which then alter fish growth and survival through trophic interactions (e.g. Schindler et al. 2005). Traits in fishes influenced by individual growth are wide ranging, including (but not limited to) age at maturity, size at maturity, brood number (fecundity), offspring (egg) size, timing of developmental stage (e.g. migration, metamorphosis), habitat type, choice of prey, vulnerability to predators and many more (Roff 1986, 2002; Jobling 1994; Wootton 1998; Hutchings 2002). Differences between species or populations in thermal reaction norms for growth can lead to competitive exclusion by other species with a better adapted reaction norm for a given environment. For example, Arctic char (*Salvelinus alpinus*) are more energetically efficient under cold temperatures or under ice but can be competitively excluded by brown trout (*Salmo trutta*) under warmer or more nutrient-rich conditions (Finstad et al. 2011; Helland et al. 2011). Fish can respond to changes in prey availability and energetic quality by modifying their distribution at broad spatial scales in the ocean (Perry et al. 2005; Sorte et al. 2010; Pinsky et al. 2013), vertically in the water column in lakes or the ocean (Dulvy et al. 2008; Pinsky et al. 2013), within stream networks (Comte and Grenouillet 2013), or by selecting different prey (Volkov 2012).

## Other drivers of selection

Importantly, the effects of climate change on organisms will rarely act in isolation of other selection pressures. Many anthropogenic impacts drive contemporary evolution (Kinnison and Hendry 2001; Reznick and Ghalambor 2001; Stockwell et al. 2003; Hendry et al. 2008) and affect many of the same traits as climate change. Changes in age at maturity, demography and density (abundance), caused by fisheries, for example, feed back into the rate of response to climate change that we might expect because of the influence that factors such as effective population size, genetic variance and generation time have on rates of evolution (Hutchings and Fraser 2008). Changes in species composition can also generate rapid evolution (e.g. Reznick and Bryga 1987; Reznick et al. 1990; Walsh and Reznick 2011), and climate change is causing large-scale redistribution of fish communities simultaneously with human-mediated species movements (Perry et al. 2005; Dulvy et al. 2008; Comte and Grenouillet 2013).

## Evidence for potential evolutionary responses in key traits

The most compelling evidence for the potential of evolutionary responses to anthropogenic climate change originates from cases of contemporary evolution in particularly relevant traits, especially through allochronic adaptation (i.e. changes over time) in introduced species. For example, sockeye salmon (*Oncorhynchus nerka*) introduced into Lake Washington in the 1930s and 1940s have diverged in developmental rates and survival at different temperatures (Hendry et al. 1998). Recent evolution in thermal tolerance has occurred in fish exposed to thermal effluents, such as mosquitofish tested after 30 years of exposure to abnormally warm water (Meffe et al. 1995). Artificial selection can induce much faster evolution, such as enhanced cold tolerance in three-spined sticklebacks (*Gasterosteus aculeatus*) within just three generations (Barrett et al. 2011), and improved heat tolerance in rainbow trout within 15 generations (Ineno et al. 2005). Spawn timing can also respond quickly to hatchery selection: a 2-week advance followed just four generations of selection in coho salmon (*Oncorhynchus kistutch*) (Neira et al. 2006).

Numerous other relevant traits have also evolved rapidly when exposed to a new environment (Reznick and Ghalambor 2001). For example, Chinook salmon (*Oncorhynchus tshawytscha*) from a single-source population in the Sacramento Valley in California, US, introduced to New Zealand, diverged quickly from their ancestral phenotypes in many traits: size at age and age at maturity (Kinnison et al. 2011), freshwater growth rates and migration timing (Quinn et al. 2001), egg size and number (Kinnison et al. 1998).

## Evidence for climate-induced phenotypic change in fish

For this special issue, we undertook a specific literature review of phenotypic responses to climate change in wild fish populations to clarify whether impacts of climate change are evident and what is known about the mechanisms behind these responses. We assessed the methods of inference for genetic or plastic mechanisms in each paper, using the categories identified by Merilä and Hendry (this volume). They identified six methods for inferring genetic change (quantitative genetic animal models, common-garden studies, model predictions, experimental evolution, space-for-time substitution, and molecular genetics) and five methods for inferring plastic change (quantitative genetic animal models, common-garden studies, experimental evolution, fine-grained population responses and individual plasticity in nature). They further asked whether the response was adaptive and how the causal driver was inferred.

To identify papers that described a phenotypic change in a natural population and provided evidence that climate drove the phenotypic change, we searched for literature in the Web of Science in which the words climate' or ‘climatic change’ (‘climat* change’), and ‘adaptation’, ‘plasticity’ or ‘phenotypic change’, appeared in the publication title or topic area. Because these searches primarily identified papers addressing human adaptation to climate change or existing variation among populations, we refined our search by combining (‘climat* change’) with phenotypic traits we expected to be sensitive to climate (‘spawning’ or ‘spawn timing’, ‘migration timing’ or ‘migrat*’, ‘emergence timing’, ‘age at maturity’, ‘egg size’, ‘development time’ or ‘development rate’). Finally, we combined ‘adaptation’ with common names of fishes that have been well studied (stickleback, perch, bass, char, smelt, herring, pollock, cod and salmon). Although this is not an exhaustive list of all possible search combinations, it encompasses a broad cross section of the available literature.

Most of the papers that initially appeared relevant instead described (i) existing heterogeneity among populations (e.g. along spatial climatic gradients) that presumably reflects adaptation to different environments, (ii) experimental exposure to conditions predicted with climate change, such as elevated temperature or lower pH or (iii) changes in abundance or recruitment and thus did not satisfy our criteria. Because our results might appear to be biased towards salmonids, we made a concerted effort to obtain evidence of phenotypic change in other fish taxa by thoroughly checking major reviews of fish responses to climate change in both freshwater and marine environments (e.g. Roessig et al. 2004; Ficke et al. 2007; Graham and Harrod 2009; Staudinger et al. 2012; Griffiths 2013), and taxonomically broader reviews of evolutionary responses to climate change (e.g. Hendry and Kinnison 1999; Kinnison and Hendry 2001; Stockwell et al. 2003; Parmesan 2006; Carroll et al. 2007; IPCC 2007). In sum, we believe that our results reflect broader patterns in the literature on observed, natural fish responses to climate change.

**Figure 3:**
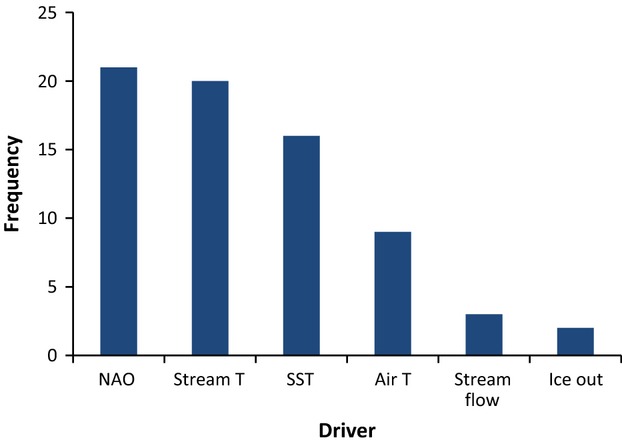
Frequency distribution of environmental drivers correlated with phenotypic change. NAO, North Atlantic Oscillation, *T*, temperature, SST, sea surface temperature; ice out is the day when a lake is free of all ice. We grouped counts by the species within a reference.

We found 30 papers that generally fit our criteria. These papers examined 11 traits (Fig. 2) in 26 species (Table 1). Attrill and Power (2002) examined six additional species (*Trisopterus luscus, Trisopterus minutus, Pomatoschistus* spp.*, Anguilla anguilla, Agonus cataphractus*, and *Syngnathus rostellatus*) in which they found significant correlations between the NAO and abundance but not growth. Most reports of phenotypic change described shifts in reproductive phenology (*N* = 17 adult migration timing and *N* = 10 spawn timing; Table 1). Changes in growth and juvenile size (*N* = 17), age at maturity (*N* = 5), age at seaward migration/smolting (*N* = 2) and fecundity (*N* = 2) were also reported. The distribution of studied taxa was biased towards salmon (especially Atlantic salmon [*Salmo salar*], but also Pacific salmon [*Oncorhynchus* spp.]) and Atlantic cod. All studies attributed phenotypic change to temperature variation, either through water temperature measurements directly, or air temperature and the NAO, ice break-up dates, temperature-driven changes in prey abundance or stream flow (Fig. 3). Geographically, the studies included North America and Europe, with most marine reports being from the North Atlantic (Table 1).

## Evolutionary mechanisms postulated or demonstrated

Only one paper (Kovach et al. 2012) utilized molecular genetic data to document a shift in genotype frequencies associated with a shift in phenotypes (Table 2). Pink salmon (*O. gorbuscha*) in Auke Creek, Alaska, historically maintained a bimodal distribution in migration timing, with the early and late migrants about 3 weeks apart. A putatively neutral genetic marker was experimentally inserted into late-migrants in 1979; marker frequencies were stable from 1981 to 1989 and clearly differentiated early and late migrants. The two segments of the run had distinct morphological traits and maturation schedules, and genetic data showed little gene flow between them. Kovach et al. (2012) tracked the frequency of this late-migrant marker compared with other markers from 1983 to 2011. The late-migrant marker decreased rapidly in the late 1980s or early 1990s, and the proportion of the run exhibiting the late-migration phenotype has remained very low since then. Incidentally, this was near the peak of a PDO cycle (Fig. 1).

**Table 2:** List of studies of phenotypic trends in response to climate

Species	Location	Trait	Genetic	Plastic	Adaptive	Causality	Reference
American shad (*Alosa sapidissima*)	Columbia River, US	Migration timing (A)	–	Yes (1)	–	Yes (1, 2)	Quinn and Adams (1996)
Atlantic salmon (*S. salar*)	Norway	Age at maturity	–	Yes (1)	–	Yes (1, 2)	Otero et al. (2012)
Atlantic salmon (*S. salar*)	Norway	Age at maturity	–	Yes (1)	–	Yes (1, 2)	Jonsson & Jonsson (2004)
Atlantic salmon (*S. salar*)	Scotland	Age at maturity	–	Yes (1)	–	Yes (1, 2)	Todd et al. (2012)
Atlantic salmon (*S. salar*)	31 stocks N. Am & Eur	Age at smolting	–	–	–	Yes (1, 2)	Russell et al. (2012)
Atlantic salmon (*S. alar*)	Scotland	Age at smolting	–	Yes (1)	–	Yes (1, 2)	Todd et al. (2012);
Atlantic salmon (S. salar)	Scotland & Canada	Growth	–	Yes (1)	–	Yes (1, 2)	Friedland et al. (2005)
Atlantic salmon (*S. salar*)	NE Atlantic	Growth	–	Yes (1)	–	Yes (1, 2)	Todd et al. (2008)
Atlantic salmon (*S. salar*)	Dalälven River	Migration timing (A)	–	Yes (1)	–	Yes (1, 2)	Dahl et al. (2004)
Atlantic salmon (*S. salar*)	Asturian Rivers, Spain	Migration timing (A)	–	Yes (1)	–	Yes (1, 2)	Valiente et al. (2011)
Atlantic salmon (*S. salar*)	NE US & SE Canada	Migration timing (A)	–	Yes (1)	–	Yes (1, 2)	Juanes et al. (2004)
Atlantic salmon (S. salar)	Northern Ireland	Migration timing (J)	–	Yes (1)	–	Yes (1, 2)	Kennedy and Crozier (2010)
Atlantic salmon (*S. salar*)	62 stocks N. Am & Eur	Migration timing (J)	–	–	–	Yes (1, 2)	Russell et al. (2012)
Bass (*Dicentrarchus labrax*)	Thames estuary, UK	Size	–	Yes (1)	–	Yes (1, 2)	Attrill and Power (2002)
Bream (*Abramis brama*)	Estonia	Spawn timing	–	–	–	Yes (1, 2)	Ahas and Aasa (2006)
Bream (*Abramis brama*)	Estonia	Spawn timing	–	–	–	Yes (1, 2)	Noges and Jarvet (2005)
Brown Trout (*S. trutta*)	Dalalven River	Migration timing (A)	–	Yes (1)	–	Yes (1, 2)	Dahl et al. (2004)
Cod (*G. morhua*)	North Atlantic	Age at maturity	–	Yes (1)	–	Yes (1, 2)	Ottersen et al. (2006)
Cod (*G. morhua*)	Barents Sea	Fecundity	–	Yes (1)	–	Yes (1, 2)	Kjesbu et al. (1998)
Cod (*G. morhua*)	Arcto-Norwegian region	Fecundity	–	Yes (1)	–	Yes (1, 2)	Sundby and Nakken (2008)
Cod (*G. morhua*)	Gulf of Alaska	Growth	–	Yes (1)	–	Yes (1, 2)	Hurst et al. (2012)
Cod (*G. morhua*)	Barents Sea	Juvenile survival	–	Yes (1)	–	Yes (1, 2)	Beaugrand et al. (2003)
Cod (*G. morhua*)	Norway	Size	–	Yes (1)	–	Yes (1, 2)	Rogers et al. (2011)
Coho salmon (*O. kisutch*)	Auke Creek, Alaska	Migration timing (J,A)	–	Yes (1)	–	Yes (1, 2)	Kovach et al. (2013)
Cutthroat trout (*O. clarkii clarkii*)	Auke Creek, Alaska	Migration timing (FW to S)	–	Yes (1)	–	Yes (1, 2)	Kovach et al. (2013)
Dab (*Limanda Limanda*)	Thames estuary, UK	Size	–	Yes (1)	–	Yes (1, 2)	Attrill and Power (2002)
Dolly Varden char (*S. malma*)	Auke Creek, Alaska	Migration timing (FW to S)	–	Yes (1)	–	Yes (1, 2)	Kovach et al. (2013)
Eurasian dace (*Leuciscus cephalus*)	Estonia	Spawn timing	–	–	–	Yes (1, 2)	Ahas and Aasa (2006)
Eurasian ruffe (*G. cernua*)	Estonia	Appearance	–	No (1)	–	Yes (1, 2)	Ahas and Aasa (2006)
Eurasian ruffe (*G. cernua*)	Estonia	Spawn timing	–	–	–	Yes (1, 2)	Ahas and Aasa (2006)
European perch (*Perca fluviatilis*)	Estonia	Appearance	–	No (1)	–	Yes (1, 2)	Ahas and Aasa (2006)
European perch (*Perca fluviatilis*)	Estonia	Spawn timing	–	–	–	Yes (1, 2)	Ahas and Aasa (2006)
Flounder (*Platichthys flesus*)	UK	Migration timing (A)	–	Yes (1)	–	Yes (1, 2)	Sims et al. (2004)
Flounder (*Platichthys flesus*)	Thames estuary, UK	Size	–	Yes (1)	–	Yes (1, 2)	Attrill and Power (2002)
Herring (*Clupea harengus*)	Thames estuary, UK	Growth	–	Yes (1)	–	Yes (1, 2)	Attrill and Power (2002)
Northern pike (*Esox lucius*)	Estonia	Spawn timing	–	–	–	Yes (1, 2)	Ahas and Aasa (2006)
Pink salmon (*O. gorbuscha*)	Auke Creek, Alaska	Migration timing (A)	Yes (1)	–	–	Yes (1, 2)	Kovach et al. (2012)
Pink salmon (*O. gorbuscha*)	Auke Creek, Alaska	Migration timing (A)	–	Yes (1)	–	Yes (1, 2)	Taylor (2008)
Pink salmon (*O. gorbuscha*)	Auke Creek, Alaska	Migration timing (J)	–	Yes (1)	–	Yes (1, 2)	Taylor (2008)
Pink salmon (*O. gorbuscha*)	Auke Creek, Alaska	Migration timing (J,A)	–	Yes (1)	–	Yes (1, 2)	Kovach et al. (2013)
Plaice (*Pleuronectes platessa*)	North Sea	Growth	–	Yes (1,2)	–	Yes (1, 2)	Teal et al. (2008)
Plaice (*Pleuronectes platessa*)	Thames estuary, UK	Size	–	Yes (1)	–	Yes (1, 2)	Attrill and Power (2002)
Roach (*Rutilus rutilus*)	Lake Geneva, France	Ovary devpmt, spawn timing	–	Yes (1)	–	Yes (1, 2)	Gillet and Quétin (2006)
Roach (*Rutilus rutilus*)	Estonia	Spawn timing	–	–	–	Yes (1, 2)	Noges and Jarvet (2005)
Smelt (*Osmerus eperlanus*)	Thames estuary, UK	Growth	–	Yes (1)	–	Yes (1, 2)	Attrill and Power (2002)
Smelt (*Osmerus eperlanus*)	Thames estuary, UK	Size	–	Yes (1)	–	Yes (1, 2)	Attrill and Power (2002)
Smelt (*Osmerus eperlanus*)	Estonia	Spawn timing	–	–	–	Yes (1, 2)	Ahas and Aasa (2006)
Sockeye salmon (*O. nerka*)	Fraser River, Canada	Age at maturity	–	Yes (1)	–	Yes (1, 2)	Cox & Hinch (1997)
Sockeye salmon (*O. nerka*)	Columbia River, US	Migration timing (A)	Yes (2)	Yes (1)	Yes (1)	Yes (1, 2)	Crozier et al. (2011)
Sockeye salmon (*O. nerka*)	Columbia River, US	Migration timing (A)	–	No (1)	–	Yes (1, 2)	Quinn and Adams (1996)
Sockeye salmon (O. nerka)	Auke Creek, Alaska	Migration timing (J,A)	–	Yes (1)	–	Yes (1, 2)	Kovach et al. (2013)
Sockeye salmon (*O. nerka*)	SW Alaska	Size	–	Yes (1)	–	Yes (1, 2)	Schindler et al. (2005)
Sole (*Solea solea*)	North Sea	Growth	–	Yes (1,2)	–	Yes (1, 2)	Teal et al. (2008)
Sole (*Solea solea*)	Thames estuary, UK	Size	–	Yes (1)	–	Yes (1, 2)	Attrill and Power (2002)
Sprat (*Sprattus sprattus*)	Thames estuary, UK	Growth	–	Yes (1)	–	Yes (1, 2)	Attrill and Power (2002)
Walleye (*Sander vitreus*)	Minnesota, US	Spawn timing	–	Yes (1)	–	Yes (1, 2)	Schneider et al. (2010)
Whiting (*Merlangius merlangus*)	Thames estuary, UK	Growth	–	Yes (1)	–	Yes (1, 2)	Attrill and Power (2002)

The columns identify whether a genetic or plastic basis for the trait was identified (Yes, No, or – = not tested explicitly), and by what method (Genetic: 1 = Molecular genetic methods, 2 = Comparison to model predictions; Plastic: 1 = Fine-grained population response, 2 = Experimental studies). If the study tested whether the response was adaptive, it is indicated in the next column (1 = phenotypic selection estimates). All studies attributed causality to environmental factors through regression analysis and reference to other work (Causality = Yes, 1 = Common sense or existing knowledge, 2 = phenotype-environment correlations). Species genera: S. *Salmo*, O. *Oncorhynchus, G. cernua: Gymnocephalus, G. morhua: Gadus*. Trait: A, adult, J, juvenile, FW to S, freshwater to saltwater migration.

Rapid changes occurred in the late-migrant locus, but not numerous microsatellite loci, indicating that natural selection caused the shift rather than genetic drift. However, the phenotypic target of selection is not entirely clear. High temperatures occurred during the years of rapid allele frequency change, and the early-migrating phenotype appears to have adaptations to warm temperature at multiple life stages (Fukushima and Smoker 1997; Smoker et al. 1998). Kovach et al. (2012) noted that the loss of the late-migrating phenotype was sudden and apparently due to selection against them, but that the more gradual trend in the median migration timing was consistent with other plastic drivers of migration date. Historically, marine survival was lower in the early-migrating fish, suggesting that the shift in adult migration timing might have negative consequences at other life stages. However, the adaptiveness of ongoing phenotypic change requires further testing.

Several other studies have explored climatic drivers of selection on sockeye salmon, which appears to be more likely to respond evolutionarily to certain pressures than other species. Quinn and Adams (1996) contrasted the responses of sockeye salmon with American shad (*Alosa sapidissima*) migration timing through a shared river basin, the Columbia River. They predicted shad would employ plasticity to respond to river conditions because of the high predictability and short-time interval between adult migration timing and larval emergence, which is presumably the target of selection. Consistent with this hypothesis, they documented a very fast shift in migration timing in shad and high interannual correlation with temperature (a fine-grained population response). However, this response was faster than the cue they had postulated as the driver (river temperature), suggesting they might not have identified the full cue for the response. Quinn and Adams (1996) postulated that, unlike shad, sockeye would rely more on a genetically determined migration date because of their relatively long larval incubation time and, hence, a lack of correlation between adult and juvenile environmental conditions. Consistent with this hypothesis, they found that sockeye lagged behind the rate of temperature change and responded much more slowly than shad. Thus, the mode of inference was through phenotypic-environment correlations, and no genetic analyses or direct tests of the adaptive nature of the response were conducted. Nonetheless, extensive corollary evidence (Naughton et al. 2005; Keefer et al. 2008) that high migration temperatures reduce survival in Columbia River sockeye salmon supports the hypothesis that elevated river temperature is the primary driver of the response.

Crozier et al. (2011) followed up Quinn and Adams' (1996) paper with a more specific model of selection pressure on sockeye salmon. Crozier et al. (2011) used a functional relationship between river temperature and survival, based on individually-tracked migrating cohorts, to estimate the annual selection pressure experienced by the population. They calculated a selection differential for each year since 1949 by reconstructing fish exposure using daily migration counts and temperature measurements at dams. Building on a method pioneered by Swain et al. (2007) study of fisheries-induced evolution in cod, they then used this annual selection differential to predict the shift in mean return migration timing of offspring. They allowed plastic drivers of migration timing, including river flow, a direct (within-year) effect of temperature, and oceanic factors such as the PDO and the North Pacific Gyre Oscillation (NPGO, Di Lorenzo et al. 2008) to modify the mean expected migration timing as plastic effects. Through model selection in a state-space modelling framework, they found very strong support for including the selection differential as a predictor of migration timing; none of the alternative plastic drivers tested could explain the observed shift in migration timing nearly as well.

The model results indicated that plasticity for migration timing in this sockeye population is largely a function of river flow and that the intercept for the norm of reaction has shifted by 3–6 days over 60 years (15 generations). Thus, this approach utilized both inferential evidence for genetic change (i.e. comparing model predictions with observed phenotypic change) and inferential evidence for plastic change (i.e. a fine-grained population response). The analysis of phenotypic selection estimates supports the hypothesis that the change was adaptive, and phenotype-environment correlations and comparison of alternative drivers point specifically to climate as the selective force. The advantage of this approach over strictly genetic methods is the strong link between the purported driver and target of selection, and response of the population.

One final sockeye study (Carlson and Quinn 2007) empirically estimated selection differentials over a decade and linked the selection directly to environmental conditions (lake level). They demonstrated strong links between climate and selection on a phenotypic trait (body size). Although the environmental trends during that study were too short to demonstrate a persistent response to climate change, Carlson and Quinn (2007) presented a compelling argument that evolution in this trait is likely to occur under climate scenarios of declining July precipitation and warming lake temperature. Other methods, such as the Price equation (Boutin and Lane, this special issue, and Price 1970, 1972; Coulson et al. 2010), cannot be applied to fish populations at this point. Although animal models are beginning to be used in fish populations (Neira et al. 2006; Serbezov et al. 2010; Debes et al. 2013), they are typically only practical under hatchery conditions or in highly constricted populations where nearly all the fish can be handled.

## Plastic responses to recent climate variability or change

The remaining papers documented correlations between phenological change and environmental drivers. A strong statistical relationship at an annual time step is consistent with a plastic response rather than an evolutionary response (Merilä and Hendry 2014, this volume). Of course, correlations alone do not establish a causal link with the driver because many environmental and other factors are correlated with each other. However, independent studies demonstrating plasticity in the trait as a function of temperature, or very high resolution responsiveness, are compelling in some cases.

## Migration/spawn timing

Several papers found that migration timing in juvenile salmon has advanced at a rate similar to that of water temperature (Kennedy and Crozier 2010; Russell et al. 2012; Todd et al. 2012). The relatively short time frame (<35 years), combined with independent evidence that temperature is a strong proximate cue for smolt migration, suggests that these responses are mostly plastic. Each of these papers expressed concern that the response was maladaptive. Kennedy and Crozier (2010) showed a correlation between migration timing and marine mortality and concluded that the trend towards earlier migration has increased marine mortality because of a mismatch between river and ocean temperatures. Russell et al. (2012) and Todd et al. (2012) argued that the smaller size of younger smolts lowered their marine survival and hence could be maladaptive. However, they did not formally test for adaptiveness. Juanes et al. (2004) found that long-term trends in migration timing of Atlantic salmon are consistent with a temperature response. Most of this shift occurred almost immediately upon transplantation from a more northern stock, and thus probably represents a plastic response. They argued that the change is adaptive based on a space-for-time comparison of trends in migration timing across a latitudinal gradient associated with temperature in many populations. Ongoing changes in many populations could have an evolutionary component, although this has not been explicitly tested. Schneider et al. (2010) examined time series of ice-out and walleye (*Sander vitreus*) spawning across numerous lakes in Minnesota, USA. They found a strong correlation between ice-out and spawn timing independent from the long-term trend, indicating a probable plastic cause.

## Age at maturation

Recent work indicates that over recent decades, some Norwegian Atlantic salmon have bred at progressively older ages (1991–2005; Otero et al. 2012). The authors presented a thorough discussion of the possible mechanisms of this shift, although there is no direct evidence of a causal link between driving factors and the phenotypic change. Nonetheless, age at maturation is known to have both genetic and plastic components (Hutchings 2002). The prevailing mechanism is thought to be a threshold body size or growth rate at particular times of year, such that individuals mature only if they exceed the threshold. This threshold is genetically determined and varies among populations (Piché et al. 2008), but whether the threshold is reached in a given year depends on environmentally determined growth conditions. Thus, the immediate trend appears to be a plastic response to growth conditions.

## Growth/survival

The majority of papers in our review documented annual variation in growth or survival that correlated with temperature variation, which indicates a plastic response. Attrill and Power (2002) documented strong interannual correlations between juvenile growth rates across a wide spectrum of marine fishes that use the Thames River estuary as a nursery. This appears to be a plastic response because detrended data showed even stronger statistical relationships, indicating annual response times, which is much too short to reflect evolutionary processes. Furthermore, they suggested use of the estuary is a facultative response to growing conditions, and found similar relationships between population abundance and individual body sizes. This is inconsistent with a rapid evolutionary response, which would entail strong selection and depress population sizes, at least temporarily. Thus, the primary basis for inference is the fine-grained population response and mechanistic reasoning. Similarly, sole (*Solea solea*) and plaice (*Pleuronectes platessa*) growth rates (Teal et al. 2008), sockeye juvenile growth (Schindler et al. 2005), cod size (Rogers et al. 2011) and survival (Beaugrand et al. 2003) and fecundity (Kjesbu and Witthames 2007) are well explained by plastic, reaction-norm responses to prey quality and quantity and seasonal timing. There is no indication that these represent evolutionary responses to climate change.

## Plastic or evolutionary mechanisms

The remaining papers in our review documented coarser-grained correlations between temperature and phenotypic change, or no phenotypic change at all despite environmental change. Weak correlations could reflect either a plastic or an evolutionary response, or be coincidental. They might also reflect difficulty in identifying or procuring data on the most direct environmental driver (e.g., Pinsky et al. 2013). Nonetheless, Ahas and Aasa (2006) found that spring spawning periods have advanced significantly in three freshwater fish species (pike, *Esox lucius,* ruffe, *Gymnocephalus cernua* and bream, *Abramis brama*) and that migration timing has advanced in one species (smelt, *Osmerus eperlanus*) from 1951 to 1999, concurrently with a trend towards warmer springs. However, most of the monitored fish species exhibited no significant trends. They postulated that earlier snow melt and reduced spring flooding might have driven the observed shifts, partly because March and April weather showed the strongest correlations. One of the papers included in our review (Noges and Jarvet 2005) reported changes in spawning date over 40 years in two Estonian fish (bream and roach, *Rutilus rutilus*). They found that the former species had advanced its breeding date by 10 days, apparently tracking water temperature, but that roach spawn timing had remained constant. Roach now encounter water that is 3°C warmer during spawning than in the 1950s. This apparent lack of thermal plasticity might expose this species to selection and present opportunities for an evolutionary response over a longer time frame.

In summary, the majority of papers in our review documented relatively fine-grained population responses to temperature or snowmelt/ice-out over relatively short time frames. Although a very quick response was also documented from a single strong selection event in an extreme year (Kovach et al. 2012), as a general pattern, it provides strongest support for plastic responses to metrics of climate change. Most of these studies detrended time series prior to analysis, or otherwise removed the raw trend to isolate stochastic variation in the environmental factor as a driver of phenotypic variation. However, statistical sophistication varied. In two species (pink and sockeye salmon), the authors reported either genetic data (Kovach et al. 2012) or a pattern of phenotypic change consistent with an evolutionary response, based on a model of selection pressure (Crozier et al. 2011) or direct estimates of selection (Carlson and Quinn 2007). Most of these trends were considered adaptive for some life history stages, but all authors expressed concern that the responses might be maladaptive for subsequent life stages. None of these hypotheses regarding adaptiveness was explicitly tested. All studies linked the observed phenotypic changes to the environmental driver by phenotype-environment correlation, with or without detrending.

## Discussion

We found that despite significant long-term trends in influential environmental factors, such as ocean and freshwater temperature, and despite abundant evidence that rapid evolution in climate-sensitive traits is possible, studies of natural adaptation to climate change in fishes are rare. Most studies reported correlations between temperature and population responses at annual time steps, which are consistent with plastic responses to environmental conditions: growth, fecundity, survival, migration and reproductive phenology are all changing in concert with environmental change. Given the high level of plasticity in these traits, detecting shifts in reaction norms would require additional methods.

Whether these changes are adaptive in the context of a warming climate remains an open question. The adaptive significance of observed shifts is complicated by the existence of multiple selective pressures acting on multiple life stages. The general use of stage-specific measurements and proxies for fitness rather than lifetime fitness make detecting adaptiveness difficult. For example, although a shift in gene frequencies might reflect adaptation, the trait being measured might not be the target of selection and thus not in itself appear adaptive. For example, Kovach et al. (2012) suggested that earlier migrants had more warm-adapted phenotypes in other life stages, which increased their fitness over late migrants. If this is the case, we might expect a reversal of the trend towards earlier migration in this population because the trend towards earlier migration timing itself exposed the fish to higher temperatures. In fact, changes in emigration and spawn timing often appeared to produce a phenological mismatch. For example, in Scotland, advanced smolt emigration from rivers in response to rising stream temperature correlated with reduced marine survival (Kennedy and Crozier 2010). But the lifetime costs and benefits of these shifts are not known.

**Figure 4:**
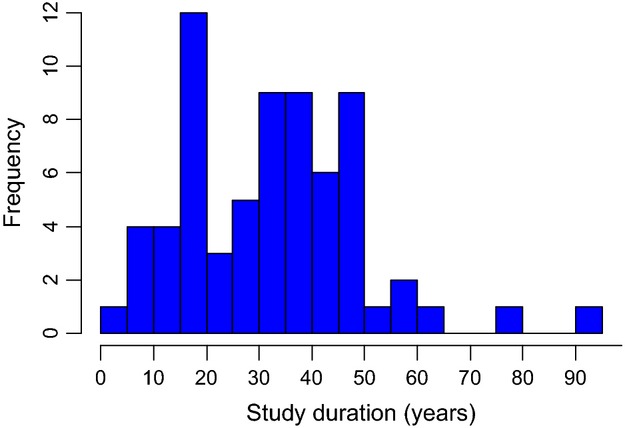
Frequency distribution of the number of years analyzed in each study.

It is clear that sufficiently high selection intensities can yield measurable selection responses in few generations in fishes. Crucial traits such as heat tolerance (Ineno et al. 2008), thermal reaction norms for growth (Kavanagh et al. 2010) and spawn timing (Neira et al. 2006) can evolve rapidly. Thus, evolution in response to climate change is certainly possible, and indeed likely, in fish. Furthermore, many studies spanned multiple generations, with the median study duration at 34 years, and the maximum at 91 years (Fig. 4). So why did so few studies document it? We propose three possible explanations.

First, the correlation-based methods generally employed are incapable of detecting the subtle shifts in reaction norms that are expected over the few generations spanned in most studies. One approach that might hold promise as a means of detecting evolutionary responses to climate change is the estimation of selection differentials over long time series (Swain et al. 2007; Crozier et al. 2011). Another might involve within-population studies of temporal shifts in the shapes of univariate or bivariate norms of reaction (Hutchings 2011). One means of detecting the latter has involved the use of probabilistic maturation reaction norms (PMRNs), albeit primarily for studies of fisheries-induced evolution (Dieckmann and Heino 2007). However, experimental studies have cautioned that temporal shifts in PMRNs can be influenced by nongenetic factors and might not always be indicative of evolution (Uusi-Heikkilä et al. 2011; Hurst et al. 2012; Díaz Pauli and Heino 2013). Research on genetic change in reaction norms could be usefully accompanied by experimental studies of selection responses by reaction norms induced by key metrics of climate change, such as temperature.

Second, natural climate variability and ‘regime shifts’ often dominated the temperature signals during study periods, rather than directional climate change (e.g. Kjesbu et al. 1998; Sundby and Nakken 2008; Rogers et al. 2011; Hurst et al. 2012). In addition to simple annual variation in climate, reversals in temperature and hence selection from decadal climate cycles might slow evolutionary responses to long-term warming and cause phenotypic trends in the opposite direction from that expected due to anthropogenic climate change (Fig. 1; Chevin 2013). These cycles can also amplify temperature trends in short-term records, but these trends do not necessarily reflect anthropogenic climate change alone. Decadal climate oscillations, such as the PDO and the NAO, cycle at 50–70 year periods. To capture evolution over these time frames is beyond the scope of most studies. In an oscillating climate, directional warming trends might impose strong selection primarily during extreme years, which will occur most often near peaks of natural decadal cycles. Kovach et al. (2012) demonstrated the potential long-term consequences of such a strong selection event quite eloquently. However, few studies will catch these exceptional events. The full evolutionary implications of natural climate cycles were not considered in any of the studies in our review.

Third, multiple selection pressures (e.g. fishing, competition from stocked fish) have the potential to overwhelm our ability to detect responses of fish to climate change. In the future, compounding threats from multiple stressors, such as hypoxia (Moran et al. 2010; Healy and Schulte 2012), high concentrations of pCO_2_ (Enzor et al. 2013) or contaminants (Terzi and Verep 2012) can lower thermal tolerance, thus increasing the likelihood that many more populations will surpass critical thermal thresholds. However, the correlation between hypoxia and temperature tolerance in a more general stress response (Anttila et al. 2013) suggests that evolution might also proceed faster than independent selection on uncorrelated traits (Etterson and Shaw 2001).

In general, the strongest evolutionary responses to climate change will likely occur in species with short generation times, subject to high or consistent selection pressure, especially near peaks of natural cycles, and in traits controlled relatively simply or with genetic variation present in existing populations. Of course adaptation, while allowing genotypes to fare better than they would have otherwise, need not translate into increased probability of persistence (Kopp and Matuszewski 2014). Many species in highly variable environments, such as the intertidal zone (Somero 2010; Tomanek 2010; Madeira et al. 2012), and many coral reef fishes are currently at or very near their thermal maxima, such that a slight increase in temperature can potentially impose strong selection (Munday et al. 2008, 2012), which might then drive populations extinct before they can adapt. However, if individual fitness tends to be higher at warm temperatures (i.e. ‘hotter is better’), then quantitative genetic models show that species in warmer environments might face lower extinction rates than those in cooler environments (Walters et al. 2012). Evolution is expected to be slower in Antarctic fishes, because they have lost functionality in relevant gene and gene regulatory areas, compared with simpler, single amino acid replacements, that would be necessary for other fish (Somero 2010; Tomanek 2010). Nonetheless, given abundant evidence that many traits in fish can respond rapidly to changes in environmentally driven selection pressures and that these traits are strongly plastic, we recommend exploration of more methods suitable for detecting temporal changes in reaction norms in fish.

## Footnotes

1 We use the term anthropogenic climate change *sensu* the United Nations Framework Convention on Climate Change as ‘a change in climate due to human activity that alters the composition of the global atmosphere and which is in addition to natural climate variability observed over comparable time periods’.
